# Ligustilide improves aging-induced memory deficit by regulating mitochondrial related inflammation in SAMP8 mice

**DOI:** 10.18632/aging.102793

**Published:** 2020-02-16

**Authors:** Wen-Li Zhu, Jia-Yi Zheng, Wei-Wu Cai, Zhao Dai, Ben-Yue Li, Ting-Ting Xu, Hao-Fei Liu, Xiao-Qi Liu, Su-Fen Wei, Yi Luo, Hong Wang, Hua-Feng Pan, Qi Wang, Shi-Jie Zhang

**Affiliations:** 1 Science and Technology Innovation Center, Guangzhou University of Chinese Medicine, Guangzhou, China; 2 Institute of Clinical Pharmacology, Guangzhou University of Chinese Medicine, Guangzhou, China; 3 Department of Neurology, The Second Affiliated Hospital of Guangzhou University of Chinese Medicine, Guangzhou, China

**Keywords:** Alzheimer’s disease, ligustilide, SAMP8 mice, mitochondrial dysfunction, neuroinflammation

## Abstract

Alzheimer’s disease (AD) is an age-related neurodegenerative disease. The main active component in *Angelica sinensis*, ligustilide, has been reported to have the protective effect on AD. Whether ligustilide could protect against age-induced dementia is still unknown. In this study, we used an aging model, SAMP8 mice to investigate the neuroprotective effect of ligustilide. The behavioral tests (Morris water maze, object recognition task, open field test and elevated plus maze) results showed that ligustilide could improve the memory deficit in SAMP8 mice. For mechanism study, we found that the protein level of P-Drp1 (fission) was decreased and the levels of Mfn1 and Mfn2 (fusion) were increased after ligustilide treatment in animals and cells. Ligustilide increased P-AMPK and ATP levels. Malondialdehyde and superoxide dismutase activity results indicated that ligustilide exerts antioxidant effects by reducing the level of oxidative stress markers. In addition, ligustilide improved neural function and alieved apoptosis and neuroinflammation. These findings have shown that ligustilide treatment improves mitochondrial function in SAMP8 mice, and improves memory loss.

## INTRODUCTION

Alzheimer’s disease (AD), also known as senile dementia, is a central nervous system degenerative disorder induced by various factors, in which senile plaques (SP), neurofibrillary wounds Knot formation and neuronal degeneration and loss are the main pathological changes [1]. Clinically, it is characterized by memory impairment, aphasia, misuse, loss of recognition, visual spatial impairment, executive dysfunction, and personality and behavioral changes such as personality dementia. However, its etiology and pathogenesis are still unclear [2]. It is widely recognized that aging process is an important foundation of AD [3]. Usually, after the age of 85, the frequency of diagnosis of AD increases to 1/3, and death occurs 3-9 years after the onset of symptoms [4, 5]. Clinical attention is increasingly paid to the prevention and treatment of AD.

*Angelica sinensis* is an herbaceous perennial plant that belongs to the Umbelliferae family [6]. Ligustilide is thought to be one of the most biologically active component in *Angelica sinensis* [7]. Most studies have shown that ligustilide can rapidly cross the blood-brain barrier and act on the central nervous system after administration [8, 9]. Ligustilide exerts anti-apoptotic and anti-oxidative effects in the nervous system [10, 11]. Recent study indicates that ligustilide could reduce memory deficits and neuronal loss in Alzheimer’s disease [12]. However, whether ligustilide could protest against aging-induced dementia is still unclear.

In this study, we employed an aging-related model, SAMP8 (senescence-accelerated mouse prone 8) [13], to explore whether ligustilide could improve cognitive dysfunction. SAMP8 is mainly characterized by age-related accelerated decline in learning and memory, and pathological changes in the central nervous system. Because of its early and natural occurrence of learning and memory impairment, SAMP8 is an excellent model for the formation of learning and memory impairment related to human aging. It is currently an ideal model for studying aging-related cognitive impairment [14]. Moreover, mice of the same progenitor strain but with normal aging process, SAMR1 (senescence accelerated mouse resistant) [15], can be usually used as an internal control when studying SAMP mice.

Therefore, this study aimed to clarify the function of ligustilide in the pathogenesis of AD. Our results showed that ligustilide could improve mitochondrial function, reduce oxidative stress and neuroinflammation.

## RESULTS

### Ligustilide improves learning and memory in SAMP8 mice

**Figure 1:**
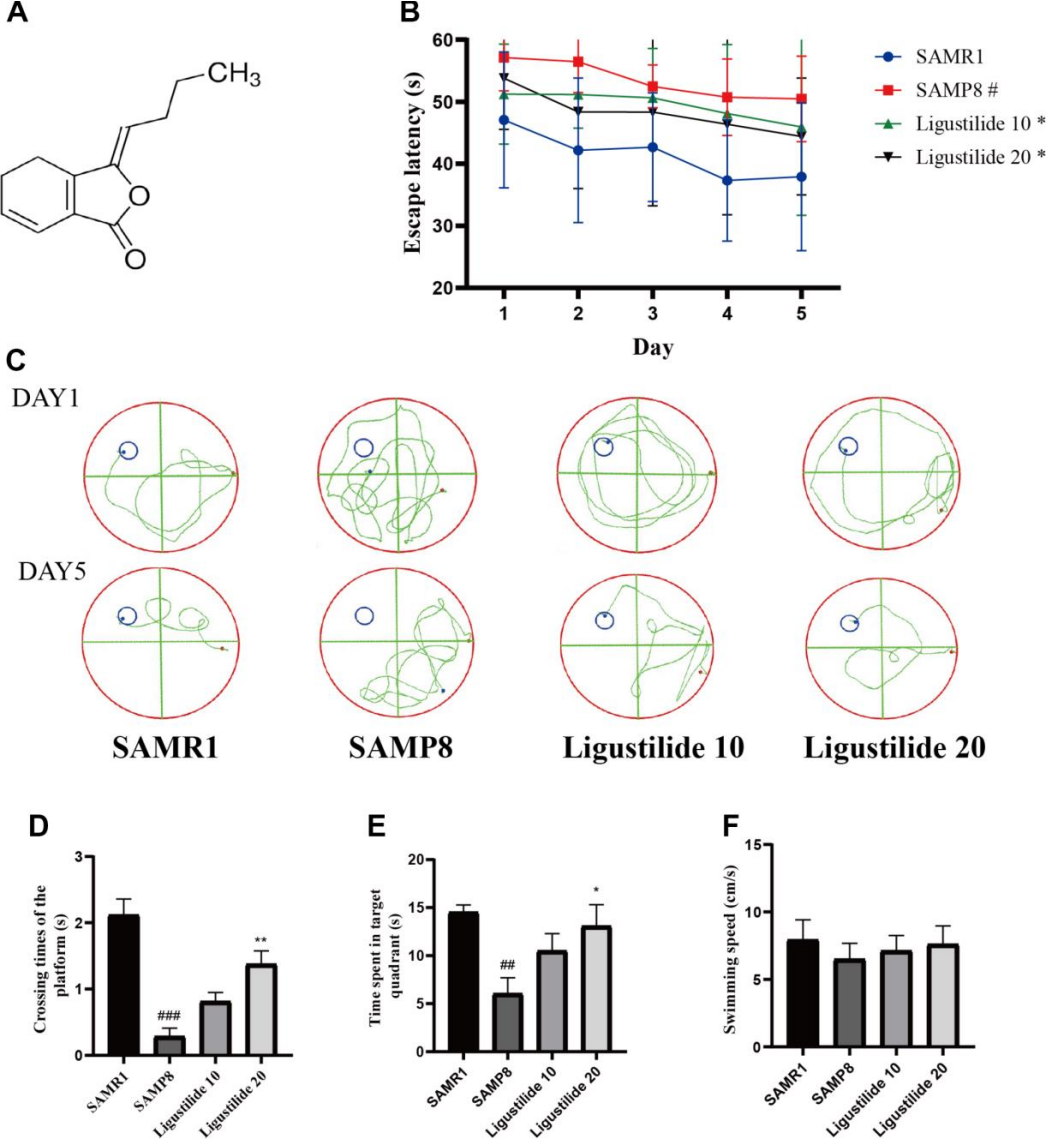
**Ligustilide improves aging-induced cognitive dysfunction in SAMP8 mice.** (**A**) The chemical structure of ligustilide. (**B**) Escape latency from five consecutive days of tests. (**C**) The swimming paths of each respective group on the sixth day. (**D**) Crossing times of the target platform in the probe trial. (**E**) Time spent in the target quadrant in the probe trial. (**F**) The swimming speed in the probe trial. Ligustilide 10 (10 mg/kg/d); Ligustilide 20 (20 mg/kg/d). Data represent mean ± SD (n = 20 per group). #*p* < 0.05, ##*p* < 0.01, ###*p* < 0.001 versus SAMR1; *p < 0.05, **p < 0.01, ***p < 0.001 versus SAMP8.

**Figure 2:**
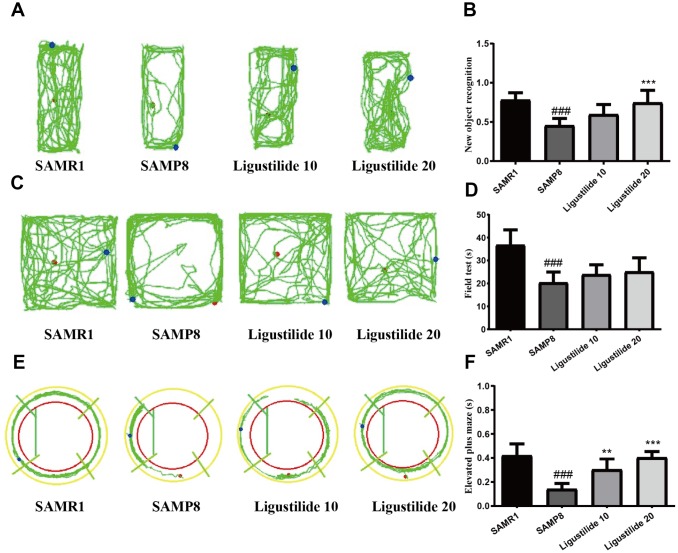
**Ligustilide improves aging-induced cognitive dysfunction in SAMP8 mice.** (**A**) Trajectory map for each respective group in the new object recognition. (**B**) Times by each respective group exploring a reference object and a new object. (**C**) Trajectory map for each respective group in the field test. (**D**) Time spent by each respective group in the field test. (**E**) Trajectory map for each respective group in the elevated plus maze. (**F**) Time spent by each respective group in the elevated plus maze. Ligustilide 10 (10 mg/kg/d); Ligustilide 20 (20 mg/kg/d). Data represent mean ± SD (n = 20 per group). #*p* < 0.05, ##*p* < 0.01, ###*p* < 0.001 versus SAMR1; *p < 0.05, **p < 0.01, ***p < 0.001 versus SAMP8.

In Morris water maze test, the time for the mice to find the hidden platform decreased gradually during the five consecutive days, and the SAMP8 group spent longer time to find the platform than the SAMR1 group (Figure 1B and 1C). Compared with the SAMP8 group, the ligustilide-administered group had a relatively short time to find the platform, and the high-dose group was shorter than the low-dose group. The crossing time of the platform and the time spent in the target quadrant showed the similar tendency (Figure 1D and 1E). The swimming speed of each group was not changed (Figure 1F). In object recognition task (Figure 2A and 2B), the time to explore new objects in SAMP8 mice was less than that in SAMR1 mice. The ligustilide group spent more time to explore new objects, when compared with SAMP8 mice. In the open field test and elevated plus maze (Figure 2C–2F), SAMP8 group showed a significant anxiety-like behavior. Ligustilide relieved this symptom.

### Ligustilide ameliorates neurodegeneration in SAMP8 mice

**Figure 3:**
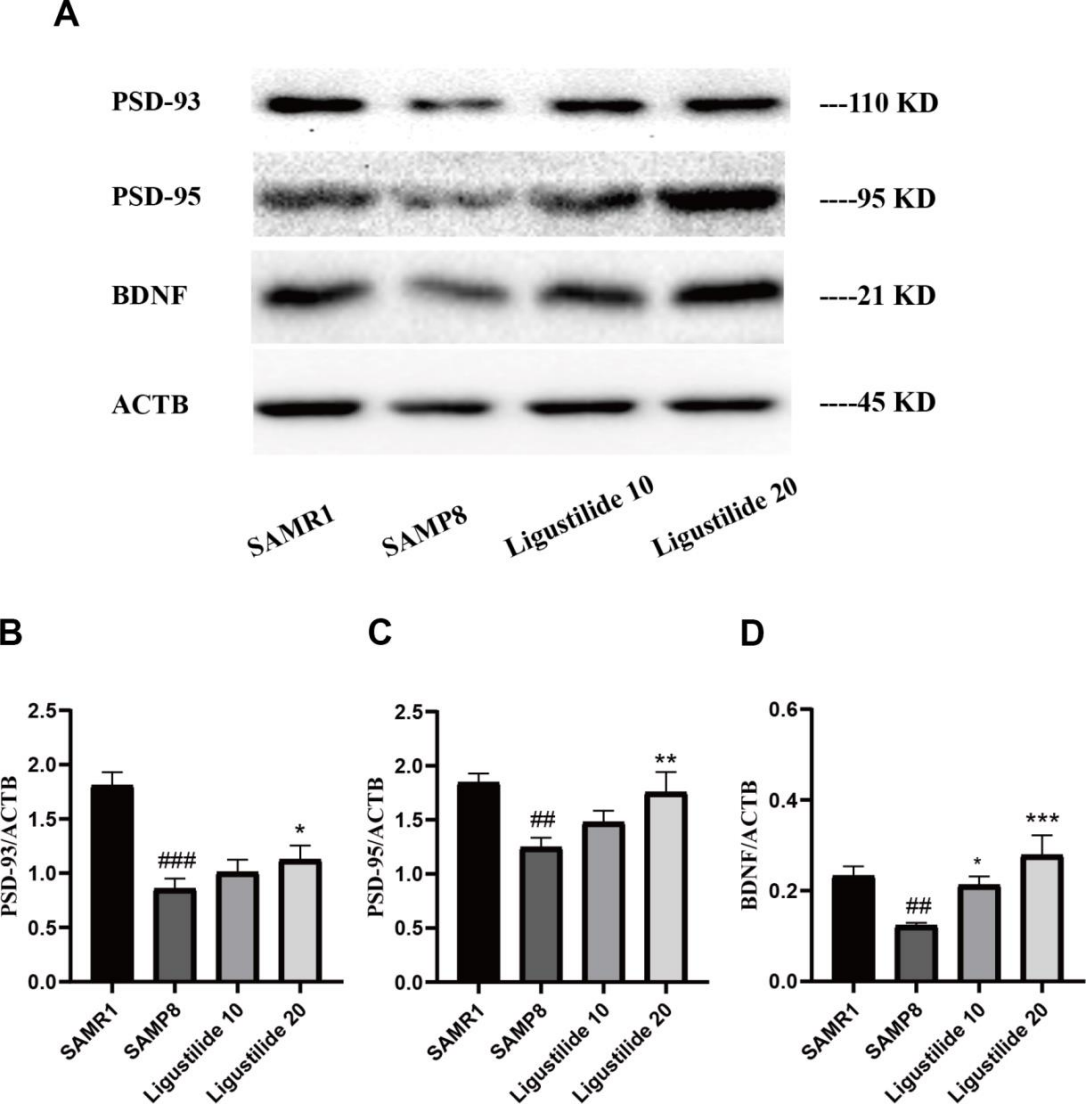
**Ligustilide ameliorates neurodegeneration in SAMP8 mice.** (**A**) Western blot analysis of (**B**) postsynapticdensity 93 (PSD93), (**C**) postsynapticdensity 95 (PSD95) and (**D**) BDNF. Ligustilide 10 (10 mg/kg/d); Ligustilide 20 (20 mg/kg/d). Data represent mean ± SD (n = 20 per group). #*p* < 0.05, ##*p* < 0.01, ###*p* < 0.001 versus SAMR1; *p < 0.05, **p < 0.01, ***p < 0.001 versus SAMP8.

As shown in Figure 3, the protein expressions of neuron-related factors, such as PSD95, PSD93 and BDNF in SAMP8 were significantly decreased. While after the treatment of ligustilide, the proteins of the high-dose group elevated to the normal level, the proteins of the low-dose group were slightly below normal. These changes indicated that ligustilide could ameliorate aging-related neurodegeneration in SAMP8 mice.

### Ligustilide improves mitochondrial function in SAMP8 mice

**Figure 4:**
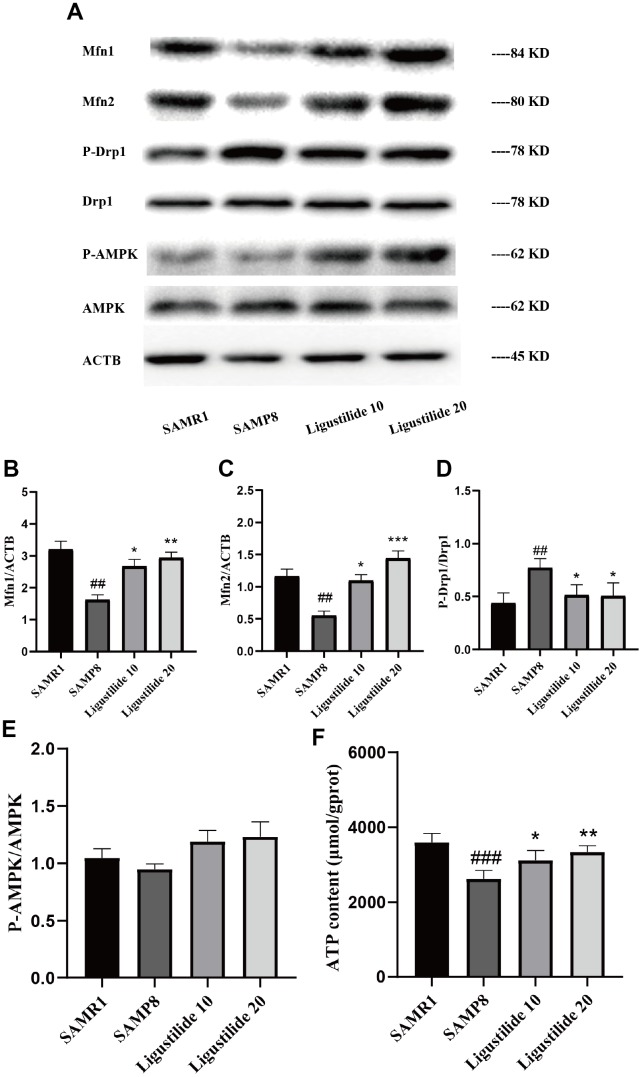
**Ligustilide ameliorates mitochondrial morphology in SAMP8 mice.** (**A**) The levels of (**B**) Mfn1, (**C**) Mfn2, (**D**) P-DRP1, DRP1, (**E**) P-AMPK and AMPK were detected in the hippocampus. (**F**) The expression of ATP was detected in the hippocampus. Ligustilide 10 (10 mg/kg/d); Ligustilide 20 (20 mg/kg/d). Data represent mean ± SD (n = 20 per group). #*p* < 0.05, ##*p* < 0.01, ###*p* < 0.001 versus SAMR1; *p < 0.05, **p < 0.01, ***p < 0.001 versus SAMP8.

Mitochondrial dysfunction is often occurred in the aging-related disease. As shown in Figure 4, the protein expression of P-Drp1 was sharply increased, while Mfn1 and Mfn2 were decreased in SAMP8. After Ligustilide treatment, the levels of Mfn1 and Mfn2 increased and P-Drp1 decreased. Ligustilide treatment relieved the fusion and fission dysregulation of mitochondria in SAMP8. Ligustilide treatment also increased the protein level of P-AMPK and the content of ATP in SAMP8 mice.

### Ligustilide attenuates H_2_O_2_-induced and Rotenone-induced mitochondrial impairment in HT22 cells

**Figure 5:**
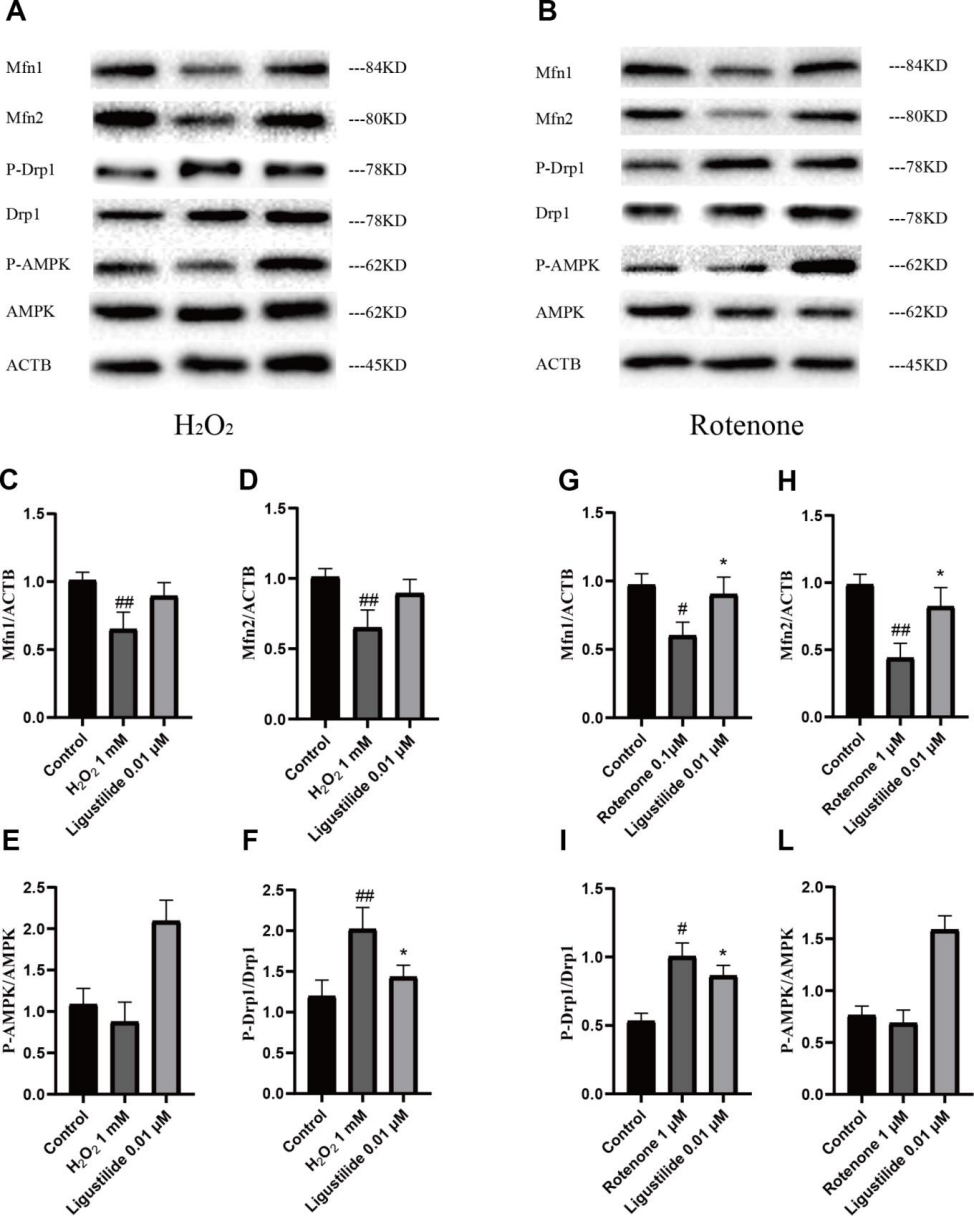
**Ligustilide attenuates H2O2-induced and Rotenone-induced mitochondrial impairment in HT22 cells.** (**A**, **B**) Western blotting was used to detect mitochondrial morphology-related proteins (**C**, **G**) Mfn1. (**D**, **H**) Mfn2. (**E**, **L**) P-Drp1. (**F**, **I**) P-AMPK.. Ligustilide 10 (10 mg/kg/d); Ligustilide 20 (20 mg/kg/d). Data represent mean ± SD (n = 20 per group). #*p* < 0.05, ##*p* < 0.01, ###*p* < 0.001 versus SAMR1; *p < 0.05, **p < 0.01, ***p < 0.001 versus SAMP8.

To determine whether ligustilide improves mitochondrial dysfunction, we employed two cell models, H_2_O_2_-exposed and Rotenone-exposed HT22 cells, to study. The H_2_O_2_ and rotenone treatment significantly reduced the intracellular Mfn1, Mfn2, and P-AMPK levels in HT22 cells, while a strongly increase in ligustilide pretreatment in HT22 cells exposed to H_2_O_2_ and rotenone (Figure 5A and 5B). In addition, as shown in Figure 5A and 5B, the intracellular level of P-Drp1, which are a major factor of maintaining mitochondrial dynamic balance, was greatly ameliorated by the pretreatment with ligustilide compared to the H_2_O_2_ only group. In conclusion, these results were consistent with in vivo data, suggesting that ligustilide pretreatment protects HT22 cells by increasing the levels of Mfn1, Mfn2, and P-AMPK and down-regulating the levels of P-Drp1.

### Ligustilide decreases oxidative stress and neuroinflammation in SAMP8 mice

**Figure 6:**
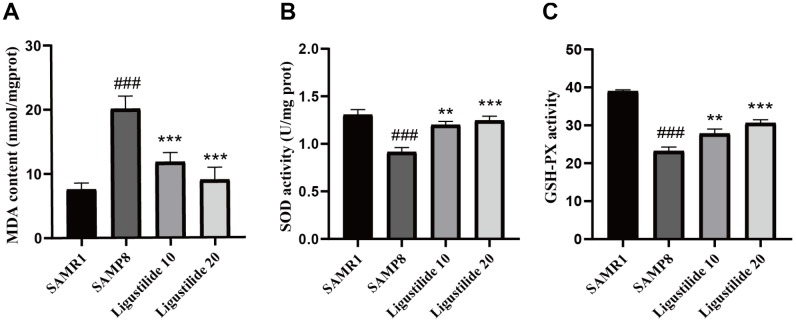
**Ligustilide reduces oxidative stress in the brains of SAMP8 mice.** The supernatant from the hippocampus homogenate was used to assay (**A**) the MDA level, (**B**) activities of SOD and (**C**) the activities of Glutathione peroxidase (GSH-PX). Ligustilide 10 (10 mg/kg/d); Ligustilide 20 (20 mg/kg/d). Data represent mean ± SD (n = 20 per group). #*p* < 0.05, ##*p* < 0.01, ###*p* < 0.001 versus SAMR1; *p < 0.05, **p < 0.01, ***p < 0.001 versus SAMP8.

**Figure 7:**
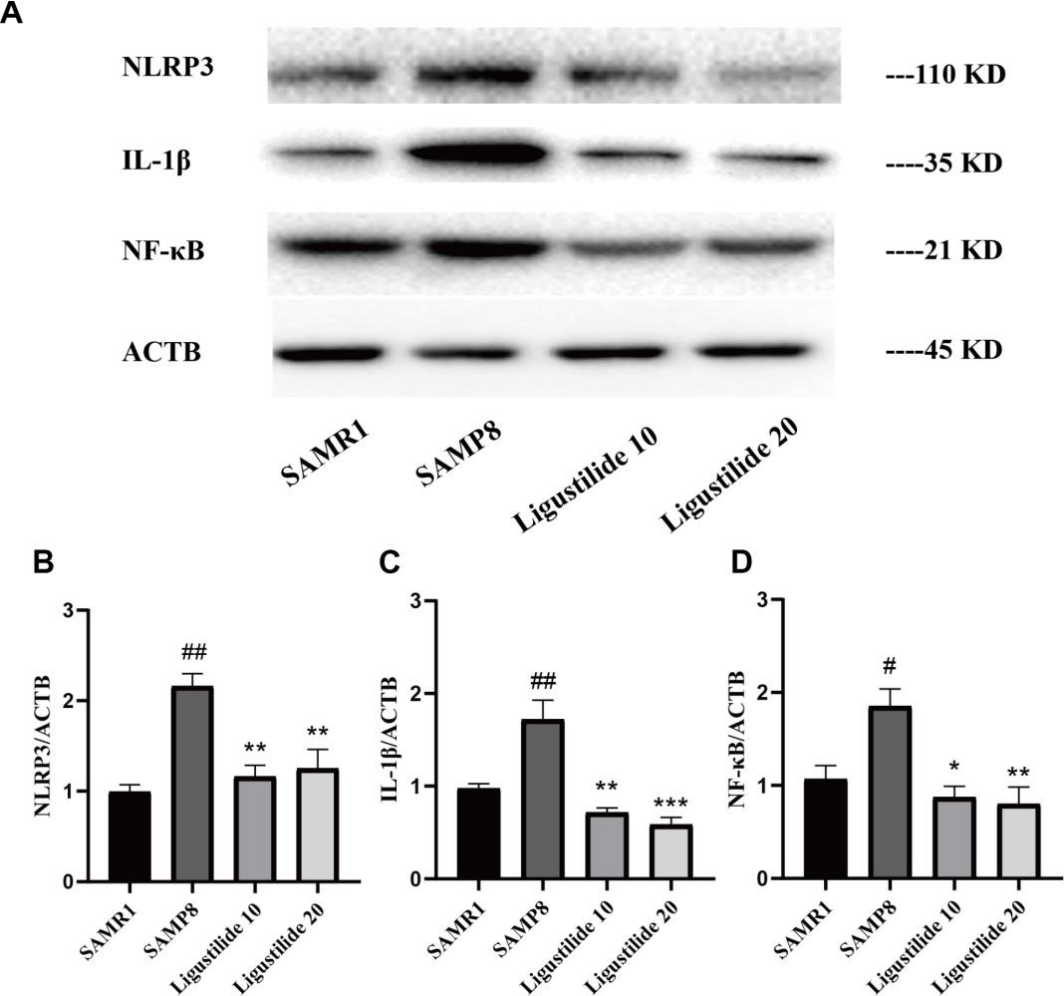
**Ligustilide reduces neuroinflammation levels.** (**A**) The levels of (**B**) NLRP3, (**C**) IL-1β and (**D**) NF-κB were detected in the hippocampus. Ligustilide 10 (10 mg/kg/d); Ligustilide 20 (20 mg/kg/d). Data represent mean ± SD (n = 20 per group). #*p* < 0.05, ##*p* < 0.01, ###*p* < 0.001 versus SAMR1; *p < 0.05, **p < 0.01, ***p < 0.001 versus SAMP8.

As shown in Figure 6, the lipid peroxidation product MDA, a marker of oxidative stress, was increased in SAMP8 mice. Ligustilide significantly reduced the level of MDA. The anti-oxidative enzymes, SOD and GSH-PX, were also detected. Ligustilide increased the activity of SOD and GSH-PX. As shown in Figure 7, the level of neuroinflammation (NLRP3, IL-1β and NF-κB) was increased in SAMP8 mice. After ligustilide treatment, these proteins were decreased almost to normal level. These results indicated that ligustilide could confrontation AD-like neuropathology in SAMP8 mice.

### Ligustilide decreases neuronal apoptosis in SAMP8 mice

**Figure 8:**
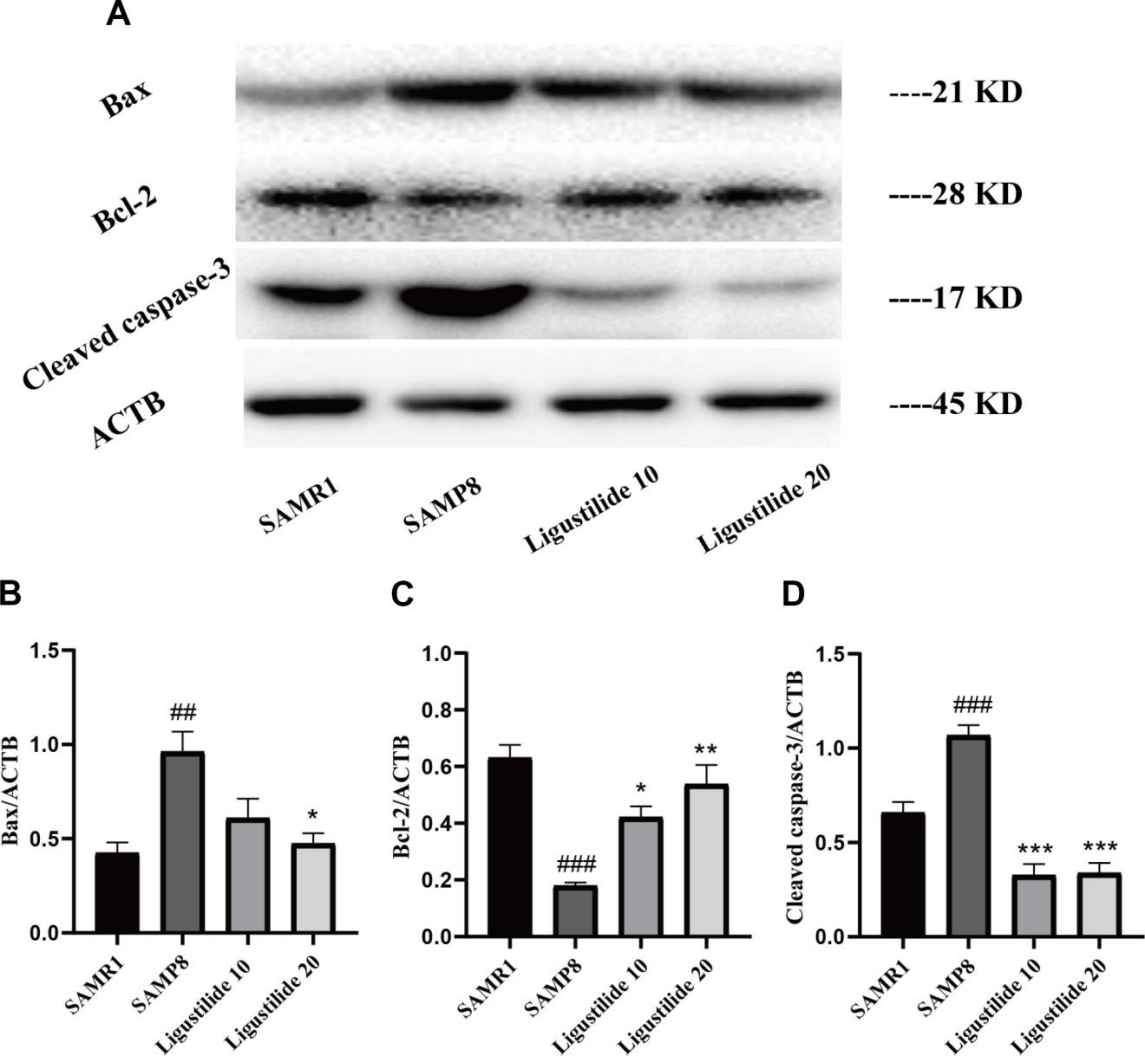
**Ligustilide protects against neuronal apoptosis in hippocampus.** (**A**) The levels of (**B**) Bax, (**C**) Bcl-2 and (**D**) Cleaved caspase-3 were detected in the hippocampus. Ligustilide 10 (10 mg/kg/d); Ligustilide 20 (20 mg/kg/d). Data represent mean ± SD (n = 20 per group). #*p* < 0.05, ##*p* < 0.01, ###*p* < 0.001 versus SAMR1; *p < 0.05, **p < 0.01, ***p < 0.001 versus SAMP8.

The expression of apoptotic markers, Bax, Bcl-2 and caspase-3, was detected to assess the apoptotic processes that occurred in all groups of tissues. As shown in Figure 8, the expression of apoptosis-related proteins Bax and cleaved Caspase-3 increased and Bcl-2 decreased in SAMP8. Ligustilide increased the Bcl-2 expression and decreased the Bax and cleaved Caspase-3 expressions.

## DISCUSSION

**Figure 9:**
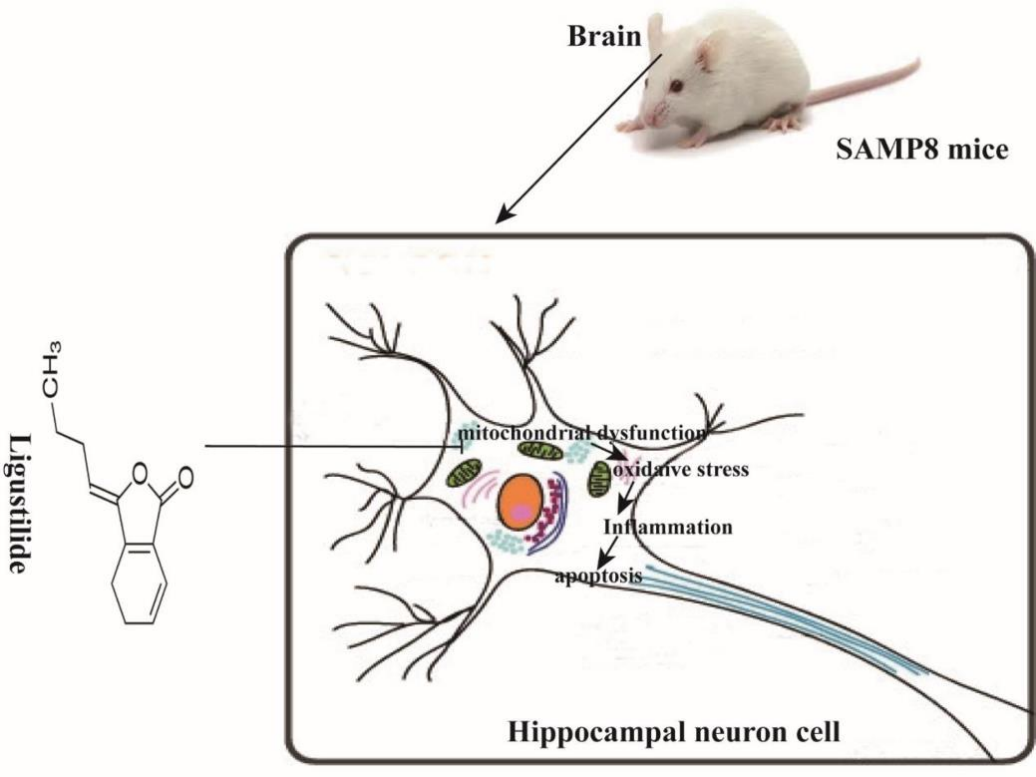
**Schematic representation of ligustilide improves aging-induced memory deficit by regulating mitochondrial-related dysfunction in SAMP8 mice.** Ligustilide improves aging-induced cognitive dysfunction in SAMP8 mice. Ligustilide ameliorates mitochondrial dysfunction in hippocampal neurons, reduces levels of oxidative stress and inflammation, reduces apoptosis in hippocampal neurons.

In this study, we demonstrate that ligustilide is capable of mitigating age-associated cognitive decline in SAMP8 mice. An eight-week-administration of ligustilide protected the learning and memory, improved mitochondrial dysfunction, reduced inflammation and apoptosis (Figure 9).

Alzheimer’s disease is a neurodegenerative disease with a variety of pathogenesis, including Cholinergic hypothesis, Amyloid beta (Aβ) hypothesis, Tau hypothesis, Excitotoxic hypothesis, Oxidative stress hypothesis, Apolipoprotein E (ApoE) hypothesis. These mechanisms eventually lead to neuronal damage or inflammation [16]. The rapid aging model mouse SAMP8 is currently recognized as an effective animal model for studying AD [17]. In this study, compared with the 10 -month-old SAMR1 mice, AD-like pathology represented by mitochondrial dysfunction, neurobehavioral deficits, oxidative stress, and inflammation were observed in the age-matched SAMP8 mice. Its behavioral and pathological features are very similar to the clinical symptoms of human AD and its complex neurological pathological changes (Xu et al. 2014). Therefore, we used SAMP8 mice as an experimental model to investigate whether Ligustilide could alleviate the pathology of AD in this study.

With the advent of aging society, the prevention and treatment of AD has become a hot topic. However, there is still no effective drug for AD treatment [18]. Recently, ligustilide has attracted widespread attention in the field of Alzheimer’s disease, owing to its capacity to reduce the levels of p-Tau, mutant APP, A*β*1-42 oligomers and monomers, ameliorate oxidative stress induced neuronal apoptosis, provide an effective neuroprotection [19, 20]. Learning and memory is an advanced function of the brain. It is an extremely complex process under the control of the central nervous system [21, 22]. In this study, we further investigated the neuroprotective effect of ligustilide on aging-related dementia.

Mitochondria are an important organelle that is ubiquitous in eukaryotic cells. It is the main site for ATP production in cells and the most important source of energy in cells. Most of the tissue cells in the human body rely on the oxidative phosphorylation of mitochondria to obtain the energy needed to maintain their own metabolism [23]. The role of mitochondria in aging was first proposed by Denham Harman in 1965. He proposed the free radical theory of aging, suggesting that with increasing age, oxygen species can attack cells and tissues and cause damage to the body. Mitochondria is one of the most important sources of reactive oxygen species [24, 25]. Since then, more and more evidence has shown that mitochondrial dysfunction causes age-related degenerative diseases, and the occurrence of mitochondrial dysfunction will increase with age, including accumulation of somatic mtDNA mutations, enhanced oxidative damage, decreased abundance and quality of mitochondria, as well as dysregulation of mitochondrial dynamics [26]. Mitochondrial dynamics means that mitochondria change their own morphology through continuous fusion and division to adapt to various stress conditions, which can meet the energy metabolism and other biological needs of cells [27]. Mitochondria are highly dynamic, their structure and function are regulated by a series of proteins involved in fission and fusion, Drp1 is involved in mitochondrial division, Mfn1 and Mfn2 are involved in mitochondrial fusion, precise regulation of mitochondrial fusion-split equilibrium helps the mitochondria to perform its normal function and ATP generation to maintain cell homeostasis [28, 29]. In the complement of Mfn1 and Mfn2, AMPK is also an energy-related signaling pathway. Due to ATP deficiency, activation of AMPK promotes mitochondrial respiration, thereby increasing mitochondrial potential [30]. In addition, AMPK activation reverses the expression of antioxidants and thus counteracts cellular oxidative stress [31]. Mitochondrial dysfunction was observed in SAMP8 mice. Since ligustilide treatment can reduce levels of Drp1 and increase levels of AMPK, Mfn1 and Mfn2 in the brain of SAMP8 mice. These results suggest that ligusrilide can protect against the mitochondrial dysfunction.

In order to further confirm the mechanisms by which ligustilide improves mitochondrial dysfunction of HT22 cells induced by H_2_O_2_ or ROT, we examined the expression of Mfn1, Mfn2, P-Drp1 and P-AMPK. In this study, H_2_O_2_ was used to induce the injury model of nerve cells, the combination of ROT and α-syn fibrils resulted in mitochondrial dynamic imbalance and leads to mitochondrial dysfunction [32]. We found that Ligustilide increase Mfn1, Mfn2 and P-AMPK, and reduce P-Drp1 in H_2_O_2_-exposed and Rotenone-exposed HT22 cells. These results again demonstrate that ligustilide can protect against the mitochondrial dysfunction.

There is increasing evidence that neuronal loss, mitochondrial mitigation and mitochondrial dysfunction occur during AD, leading to the progressive cognitive decline in AD, which may be associated with increased oxidative damage. Excessive oxidative stress can not only directly destroy cell membranes, RNA and protein, but also promote neuroinflammation and neuronal apoptosis [33]. Mitochondria are thought to be an important part of the production of reactive oxygen species in cells [34]. Oxygen is consumed in the mitochondria. When mitochondria are damaged, the body’s oxidative stress response is activated, which in turn causes apoptosis and inflammation, and the cells appear dysfunctional [35]. In this study, we find elevated levels of MDA and a decrease in GSH level and the activity of SOD in the SAMP8 mice under the treatment of ligustilide. Elevated oxidative stress was occurred in SAMP8 mice. Ligustilide effectively relieved this changes.

AD is a complex neurodegenerative disease. A variety of inflammatory factors such as IL-1β, NLRP3 and NF-κB are thought to play an important role in the pathogenesis of AD, leading to nerve damage, activity of astroglia and microglia and apoptosis [36, 37]. Glial cell hyperplasia, inflammatory cells and cytokine activation further promote or accelerate neuronal apoptosis. The apoptotic nerve cells reappear with new stimulation factors, which induce AD inflammatory reaction to occur many times and accelerate the deterioration of the disease [37]. Recent study has been demonstrated that ligustilide significantly ameliorated cognitive impairment and brain damage through antioxidant and anti-apoptotic mechanisms [38]. In our study, the level of NLRP3, IL-1β and NF-κB were decreased under the treatment of ligustilide. These results suggest that Ligustilide can protect against the inflammation.

In apoptosis, mitochondria are the center of regulation of apoptosis. Persistent mitochondrial dysfunction contributes to apoptosis due to the release of cytochrome c into the cytoplasm with subsequent activation of caspases, triggering a cascade, which initiate and carry out apoptosis [39]. The anti-apoptotic protein Bcl-2 can prevent the release of cytochrome C from mitochondria. In contrast, the pro-apoptotic protein Bax induces mitochondrial injury leading to cell death [40]. The present study showed that the apoptotic indexes Bax/Bcl-2 and cleaved Caspase-3 expressions significantly decreased after the treatment of ligustilide. These results indicated that ligustilide effectively inhibited mitochondrion-mediated apoptosis.

In conclusion, ligustilide prophylactic administration significantly improved the ability of learning and memory and brain tissue mitochondrial function in SAMP8 aging mice. Ligustilide is expected to be an effective drug for the treatment of AD in the clinic.

## MATERIALS AND METHODS

### Ethics statement

Investigation has been conducted in accordance with the ethical standards and according to national and international guidelines and has been approved by the authors institutional review board.

### Materials

Ligustilide were purchased from Chengdu Herbpurifed Co., Ltd. (purity > 98%, molecular weight: 190.24 g/mol, Figure 1A). The Malondialdehyde (MDA) assay kit and Mn-SOD and Cellular Glutathione Peroxidase Assay kit were obtained from Nanjing Jiancheng Bioengineering Institute (Nanjing, China). Antibodies mainly include Postsynapticdensity 95 (PSD95), Postsynapticdensity 93 (PSD93), NLRP3, NF-κB, IL-1β, IL-10, Bax, Bcl-2 and Caspase-3 were purchased from Cell Signaling Technology, Inc. Anti-β-actin was obtained from Sigma-Aldrich. All secondary antibodies (horseradish peroxidase-conjugated anti-rabbit IgG and mouse IgG) were purchased from Cell Signaling Technology, Inc. Other reagents were the highest quality commercial reagents.

### Animals

Male 10-month-old SAMP8 and SAMR1 mice were purchased from Beijing Vital River Laboratory Animal Technology. They were raised in The Sterility Test Lab of Guangzhou Traditional Chinese Medicine University with a 12-hour light/dark schedule, and give free access to food and water (SCXK2016-0010). All experiments were approved by the Guiding Principles for the Care and Use of Laboratory Animals that adopted and promulgated by the United States National Institutes of Health.

### Drug treatment

SAMP8 mice were randomly divided into 3 groups (n = 20), model group, ligustilide low-dose (10 mg/kg, in 3% Tween-80)), and high-dose (20 mg/kg, in 3% Tween-80). From the age of 8 months, the mice were treated daily with ligustilide. The treatments were administered once a day for 8 consecutive weeks. All drugs were administered by oral administration. Behavioral testing was started after 8 weeks.

### Behavioral testing

### Morris water maze

At the end of ligustilide treatment, the short-term working memory of mice was assessed using Morris Water Maze Test. The Morris water maze experiment was performed according to the Morris method [41]. Briefly, Mice participated in the position navigation test for five consecutive days. Mice were adapted to the fixed platform for 10 s on the first day. On the sixth day, mice were allowed to swim freely in the pool for 90 s without the original platform. The times of crossing through the original platform position, the time spent in the target quadrant and the swimming speed were used to evaluate the degree of memory consolidation.

### Object recognition task

The Object Recognition Task is a method of measuring a specific form of mice episodic memory [42]. The mice first touched two identical objects. After adapting for a period of time, one of the objects presented in the first trial will be replaced by a new one, observing the time and trajectory of the mice to distinguish between the two objects, which can indicate the episodic memory ability of mice.

### Open field test

The behavior of mice exposed to open field arena for the first time is usually used to assess the level of anxiety in mice [43]. The arena consists of a square and a camera mounted on the square. The mice were put in the box and the observer observed the changes in the behavior of the mice.

### Elevated plus maze

The elevated labyrinth is a widely accepted test method for rodent anxiety behavior. The elevated maze is a "+" shaped set-up with two closed and two open arms. The arm extends from the center platform and the center platform is raised 50-75 cm above the ground according to different protocols. For testing, the mice were placed on a central platform facing one of the closed arms.

### Brain tissue collection

At the end of the behavior test, mice were anesthetized intraperitoneal with sodium pentobarbital (30mg/kg), and perfused with PBS. The brains were quickly removed, and the hippocampus and cortex were separated from the brain. Tissues were immediately stored at -80°C until the Western blot or biochemical analyses. All of these procedures were operated on an ice-cold plate.

### MDA, SOD, and GSH-Px assays

The hippocampus tissues were homogenized with PBS and centrifuged at 12,000 × g for 10 min at 4 °C. According to the kit instructions, the supernatant was used to detect the level of MDA, the activity of SOD and GSH-Px.

### Cell culture and ligustilide treatment

Immortalized mouse hippocampal HT22 cells were maintained in the medium with 75-mL vented culture flasks in a humidified incubator at 37 °C with 5% CO_2_. The medium contained 90% DMEM medium, 10% FBS and 1% penicillin/streptomycin. 5 × 10^4^ Cells were seeded in a 96-well plates and incubated for 24 h, and 2 × 10^5^ cells were plated in 6-well plate for other experiments. Cells were pretreated with 0.01 μM ligustilide for 24 h and then cultivated with H_2_O_2_ for another 6 h before detection.

### Western blot analysis

The hippocampus tissues and HT22 cells were placed in ice-cold RIPA buffer for 15 min and 30 min, respectively. The hippocampus tissues were homogenized at 4 °C, and the supernatant was taken to prepare a sample. Protein samples were separated by SDS-PAGE analysis gel and transferred onto polyvinylidene diflouride (PVDF) membranes (Millipore). The membrane were blocked with 5 % skimmed milk for 70 min. Then the membrane was washed for three times, each time for 10 min, followed by overnight incubation at 4 °C with primary antibodies. The next day, the membrane were washed and hatched with secondary antibodies for 1 h at room temperature. Bands were scanned using an ECL chemiluminescence kit (Millipore) on a ChemiDoc MP Chemiluminescent imaging system (Bio-Rad, USA), and quantified using NIH Image J software.

### Statistical analysis

SPSS 19. 0 statistical and GraphPad Prism 5 software was used for statistical analysis of the data, one-way analysis of variance was used to analyze discrepancy between groups. Data are presented as the mean ± standard error. The level of statistically significant difference for all tests was *P* < 0.05, P<0.01.
