# Correction for: Ligustilide improves aging-induced memory deficit by regulating mitochondrial related inflammation in SAMP8 mice

**DOI:** 10.18632/aging.102998

**Published:** 2020-03-28

**Authors:** Wen-Li Zhu, Jia-Yi Zheng, Wei-Wu Cai, Zhao Dai, Ben-Yue Li, Ting-Ting Xu, Hao-Fei Liu, Xiao-Qi Liu, Su-Fen Wei, Yi Luo, Hong Wang, Hua-Feng Pan, Qi Wang, Shi-Jie Zhang

**Affiliations:** 1 Science and Technology Innovation Center, Guangzhou University of Chinese Medicine, Guangzhou, China; 2 Institute of Clinical Pharmacology, Guangzhou University of Chinese Medicine, Guangzhou, China; 3 Department of Neurology, The Second Affiliated Hospital of Guangzhou University of Chinese Medicine, Guangzhou, China

**Keywords:** correction

**This article has been corrected:** The authors requested to change the affiliation for corresponding author Shi-Jie Zhang. He is now affiliated with institution 3, instead of affiliations 1, 2, 3.

This correction does not change the content of the publication. The corrected information is provided below.

Wen-Li Zhu^1,2^, Jia-Yi Zheng^1,2^, Wei-Wu Cai^1,2^, Zhao Dai^1,2^, Ben-Yue Li^1,2^, Ting-Ting Xu^1,2^, Hao-Fei Liu^1,2^, Xiao-Qi Liu^1,2^, Su-Fen Wei^1,2^, Yi Luo^1,2^, Hong Wang^1,2^, Hua-Feng Pan^1,2^, Qi Wang^1,2^, Shi-Jie Zhang**^3^**

Original article: Aging. 2020; 12:3175–3189.  https://doi.org/10.18632/aging.102793

